# Supplementary material for: Insoluble Network Skeleton and Soluble Components of Nylon 6,6-Sputtered Nanoparticles: Insights from Liquid-State and Solid-State NMR Analysis
Source: Nanomaterials (Basel). 2024 Mar 10;14(6):497. doi: 10.3390/nano14060497 (PMC10975392; doi:10.3390/nano14060497)
Supplement: Supplementary file 1 [file nanomaterials-14-00497-s001.zip › nanomaterials-2874047-supplementary.pdf]

Supporting materials

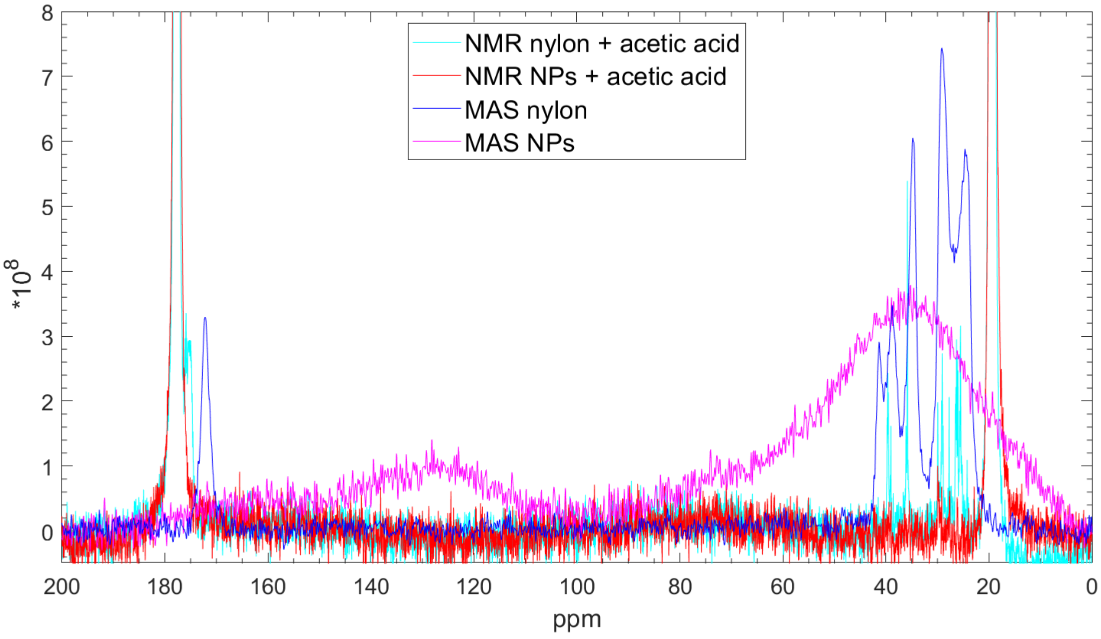

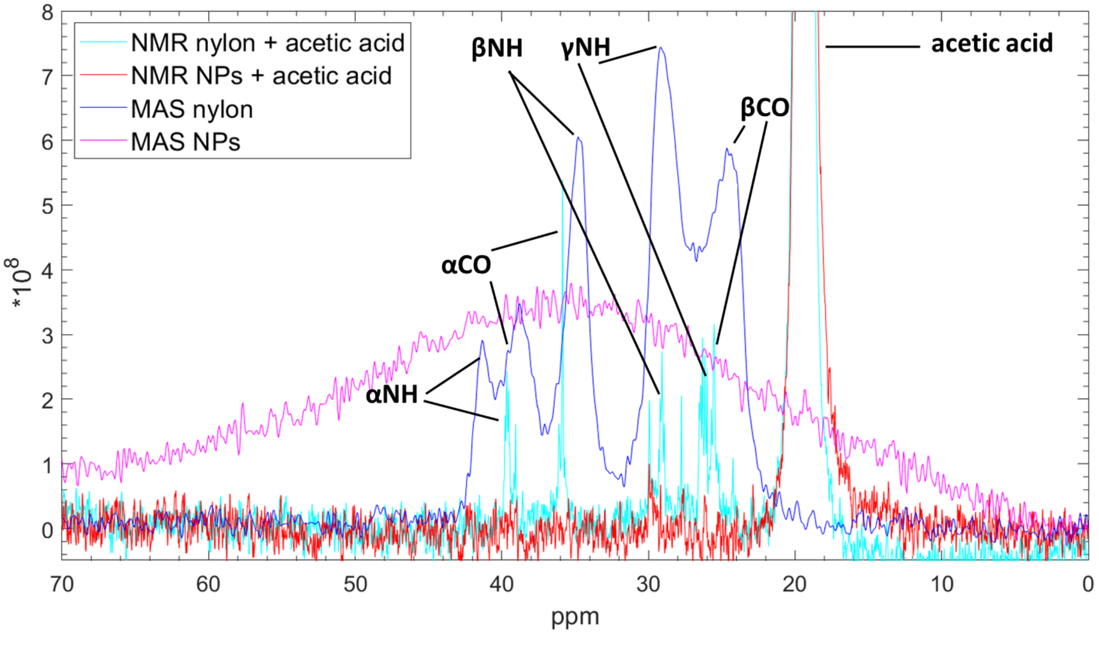

Figure S1. ^{13}C NMR spectra of the original nylon 6,6 and the NPs, measured in the solid state (MAS) and dissolved in the acetic acid (NMR). (Full spectra (above) and only aliphatic region.) It is clearly seen that in both methods we obtained well resolved spectra for the original nylon. The solid-state experiment for NPs (pink) gives wide peaks. On the other hand, the liquid state experiment, detecting only the soluble part of the NPs (red), is almost lost in the noise, although the experiment run with 157,744 scans.

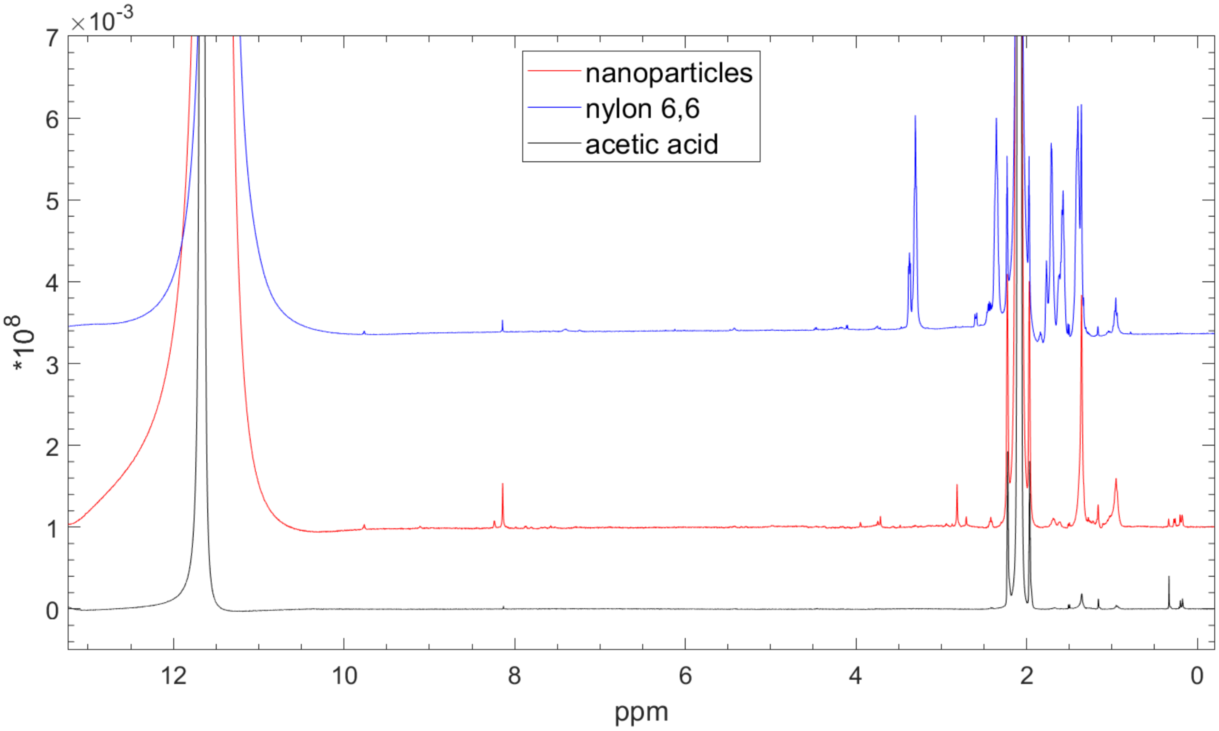

Figure S2. ^{1}H liquid NMR spectrum of the precursor nylon 6,6 and the nanoparticles N60 in acetic acid (whole spectrum). The spectrum of pure acetic acid (black) is added.

Table S1. Molar weight distributions and their ratio obtained from gel permeation chromatography (GPC) data on sputtered-nylon nanoparticles N60. M_n and M_w denote the number- and weight-average molar masses of the molecules, respectively.

	M_n [g/mol]	M_w [g/mol]	intensity of the peak
Peak 1	155	158	4
Peak 2	294	302	1
Peak 3	747	829	6

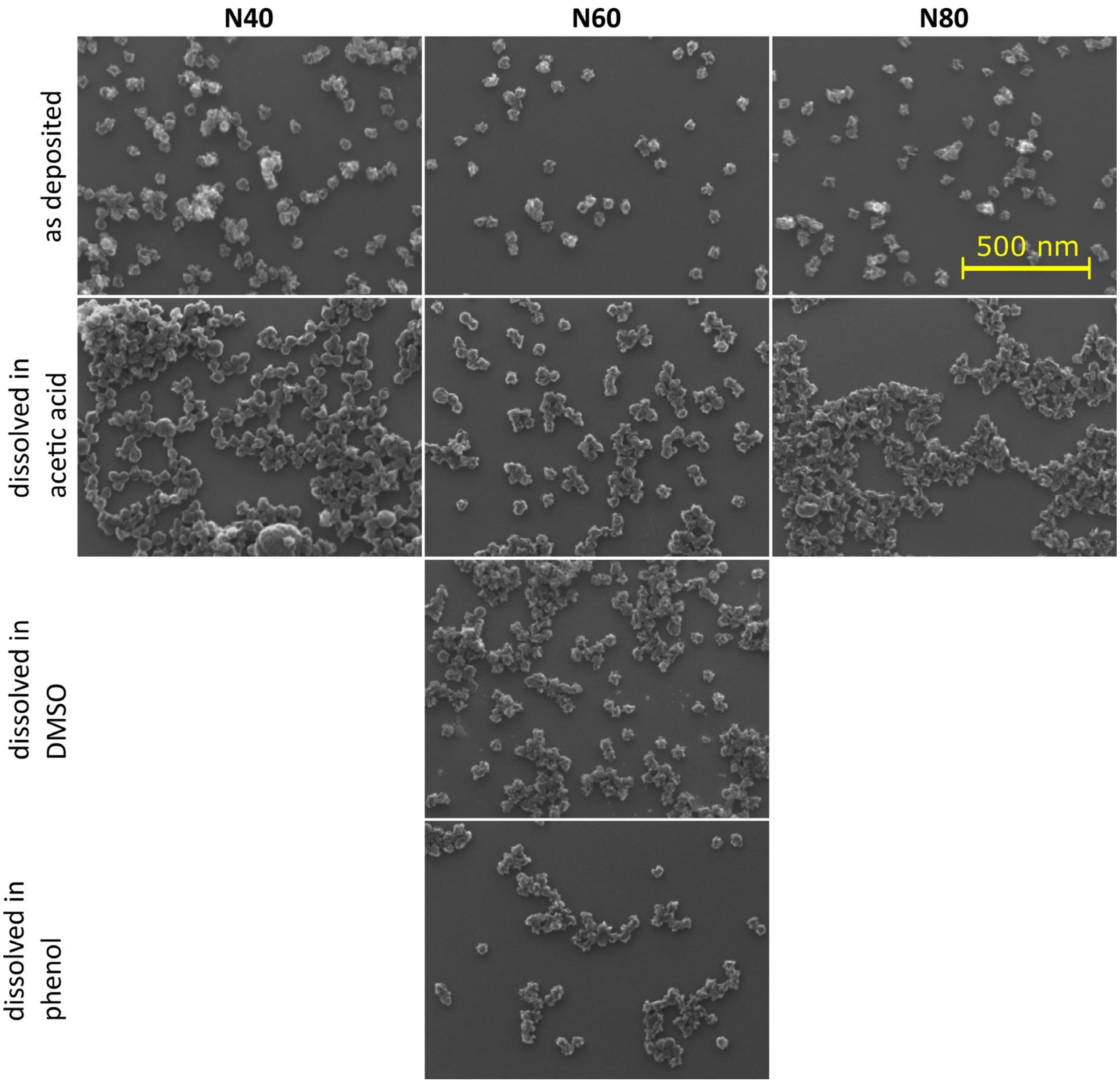

Figure S3. SEM photographs of the nanoparticles in their as-deposited state and after dissolution in three different solvents followed by drying. Nanoparticles were deposited at three different RF power levels (40 W, 60 W, and 80 W, labeled as N40, N60, and N80, respectively).

Table S2. Mean sizes of the nanoparticles from Figure S1. The measurement uncertainty of the mean size is, in all cases, about 2 nm. It is derived from the uncertainty of the measurement of a single nanoparticle and represents the uncertainty of the shift of the size distribution as a whole, rather than describing its width.

**nanoparticles**	**N40 [nm]**	**N60 [nm]**	**N80 [nm]**
as deposited	45	44	42
dissolved in acetic acid	45	46	42
dissolved in DMSO		42	
dissolved in phenol		45	

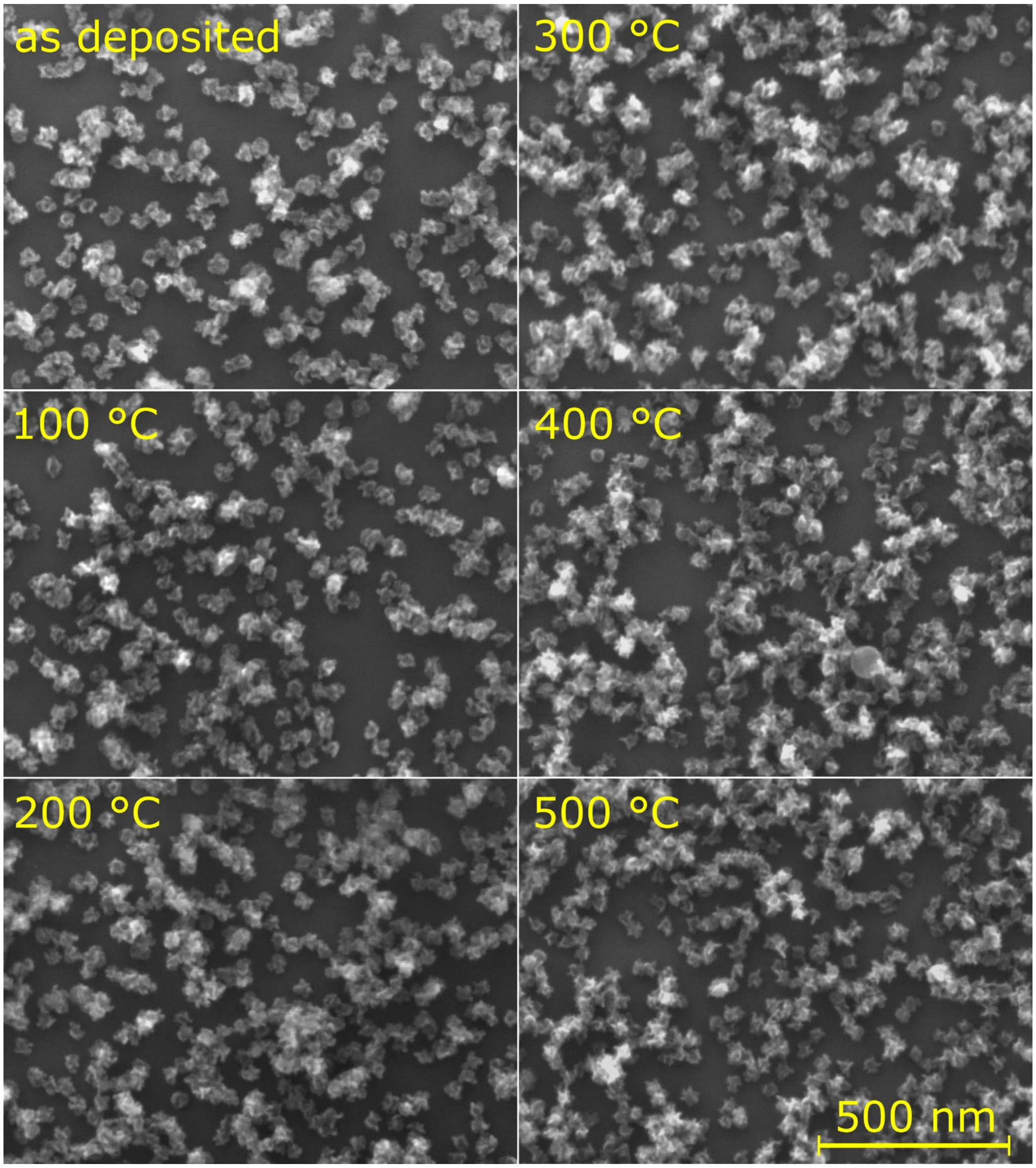

Figure S4. SEM photographs of nanoparticles deposited at RF power 60 W (N60) after heating for 20 minutes at elevated temperature indicated in the upper left corner of each image. Six identical samples were used, each for one temperature.

Table S3. Mean sizes of nanoparticles from Figure S2.

temperature	room	100 °C	200 °C	300 °C	400 °C	500 °C
diameter of N60	46 ± 4	47 ± 4	44 ± 3	42 ± 4	41 ± 4	43 ± 3
